# *Vibrio cholerae* Porin OmpU Induces Pro-Inflammatory Responses, but Down-Regulates LPS-Mediated Effects in RAW 264.7, THP-1 and Human PBMCs

**DOI:** 10.1371/journal.pone.0076583

**Published:** 2013-09-27

**Authors:** Sanica C. Sakharwade, Praveen K. Sharma, Arunika Mukhopadhaya

**Affiliations:** Department of Biological Sciences, Indian Institute of Science Education and Research (IISER) Mohali, Mohali, Punjab, India; University of Iowa Carver College of Medicine, United States of America

## Abstract

*Vibrio cholerae* porin OmpU plays a crucial role in the survival of the organism in the human gut. Various observations suggest critical involvement of OmpU in *V. cholerae* pathogenesis. However, OmpU is poorly characterized in terms of its ability to evoke cellular responses, particularly in the context of host immune system. Therefore, towards characterizing *V. cholerae* OmpU for its host immunomodulatory functions, we have studied the ability of OmpU to elicit pro-inflammatory responses in a range of immune cells which include, mouse RAW 264.7 macrophages, human THP-1 monocytes and human PBMCs. We have observed that purified OmpU induces pro-inflammatory responses in terms of production of NO, TNFα and IL-6. Interestingly, pre-treatment of the cells with OmpU suppresses the production of NO, TNFα, IL-6 as well as IL-12 upon subsequent activation with LPS. Our results therefore suggest that *V. cholerae* OmpU may have a differential regulatory role in terms of host immunomodulatory function: it can induce pro-inflammatory responses in target host immune cells, whereas it can also exert suppressive effect on LPS-induced pro-inflammatory responses. In addition, our study indicates that purified OmpU may have the ability to skew the Th1 response towards the Th2 response, presumably via suppression of IL-12 production.

## Introduction

Outer membrane of gram-negative bacteria plays a crucial role in mediating interaction between the organism and its environment. Lipopolysaccharide (LPS) and outer membrane proteins (OMPs), the structural constituents of the outer membrane, are often instrumental in bacterial pathogenesis and modulation of host cell responses. LPS and OMPs, together with other highly conserved microbial molecules, are collectively known as pathogen associated molecular patterns (PAMPs). These PAMPs are recognized by specific set of pattern recognition receptors (PRRs) present on certain host cells [1]. This PAMP-recognition event by PRRs initiates complex signaling cascades that result in activation of various components of innate immunity, of which inflammatory responses play a major role in containing the microbial infection [2].

Porins are one of the major groups of bacterial OMPs. They generally form channels across the bacterial outer membrane for solute transport. Porins perform several other functions in addition to their channel property. They are crucial for bacterial survival in harsh environments [3]. In some pathogenic strains, porins are recognized by the host immune system, and they modulate the host responses. Induction of pro-inflammatory responses and stimulation of associated cell-signaling processes have been described for various bacterial porins [4]. In addition, porins have been reported to be involved in the pathogenesis process, like host cell invasion, adherence and induction of apoptosis [5-8]. Further, porins from various gram-negative pathogenic bacteria have been considered as potential vaccine candidates. For example, porins from *Salmonella* *typhi* and Neisserial species have been reported to offer a protective effect against infection [9,10]. It has been suggested that Neisserial porin can be used as vaccine adjuvant, as it up-regulates B7-2 expression and stimulates B cells [11].

Studies by several investigators have revealed the presence of nearly six major OMPs in *Vibrio cholerae*, a gram-negative facultative enteric pathogen [12]. The expression of two porins, OmpT and OmpU, is known to be under the control of ToxR regulon, a master regulator of virulence genes in *V. cholerae*. ToxR negatively regulates *ompT*, while *ompU* is positively regulated [13]. It has been reported that *V. cholerae* OmpU provides resistance to bile acids and antimicrobial peptides. *V. cholerae* OmpU was also reported to help in adherence [14], but later studies disproved it [13]. Critical involvement of OmpU in *V. cholerae* pathogenesis is highlighted by the fact that *V. cholerae* isolates from cholera outbreaks express OmpU [15]. Importance of OmpU in pathogenesis has also been underscored in reports, which describe reduced virulence of the organism in absence of this protein [16].

As mentioned before, immunogenic and/or pathogenic properties are attributed to different gram-negative bacterial porins. However, *V. cholerae* OmpU is poorly characterized for its role in host-immunomodulation. Towards understanding the nature of OmpU in detail, we studied the effect of OmpU in RAW 264.7 murine macrophage cell line, THP-1 human monocytic cell line and human peripheral blood mononuclear cells (PBMCs). We observed that *V. cholerae* OmpU stimulated macrophage and monocytic cell lines of mouse and human origin as well as human PBMCs to produce pro-inflammatory mediators, such as TNFα, IL-6 and/or NO. Moreover, we observed that the effect of LPS in terms of production of NO, TNFα, IL-6 and IL-12 was down-modulated in cells pre-treated with OmpU. These findings suggest that OmpU plays a dual role. *V. cholerae* OmpU can induce pro-inflammatory response, while OmpU pre-treatment can suppress pro-inflammatory mediators and IL-12 response of LPS-activated cells. To the best of our knowledge this is the first report showing differential regulation of host immune responses by a porin.

## Materials and Methods

### Ethics statement

Work with human blood has been approved by the Institutional Bioethics Committee (The Bioethics Committee of IISER Mohali). Written informed consents were obtained from the donors.

### Purification of recombinant OmpU

Recombinant OmpU was purified according to the protocol of Khan et al [17]. Briefly, *E. coli* Origami B cells (EMD Millipore, Billerica, MA, USA) expressing *V. cholerae* El Tor O1 OmpU gene cloned in pET14b vector (EMD Millipore, Billerica, MA, USA) were used to isolate recombinant OmpU protein. *V. cholerae* was cultured in Luria broth (HiMedia, Mumbai, India) until the OD_600_ reached 0.5-0.6. The culture was then induced with IPTG (HiMedia, Mumbai, India) for 3 h. The cells were collected by centrifugation and the pellet was resuspended in bacterial lysis buffer (GBiosciences, St. Louis, MO, USA) containing bacterial protease inhibitor cocktail (Sigma-Aldrich, St. Louis, MO, USA). The cell suspension was subjected to ultrasonication with 30-40 pulses of 25-30 amplitude for 5 mins for cell lysis. The cell lysate was centrifuged at 18,500 xg for 30 mins at 4 °C to obtain inclusion bodies. The pellet containing inclusion bodies was washed twice with PBS containing 100 mM NaCl and was resuspended in the same buffer. The suspension was sonicated and centrifuged at 18,500 xg for 30 mins at 4 °C. The pellet was solubilized in PBS containing 8 M urea for 30-45 mins at room temperature. The solubilized pellet was centrifuged at 18,500 xg for 30 mins at 4 °C and supernatant containing the protein was supplemented with 20 mM imidazole. A manually packed Ni-NTA column (Qiagen GmbH, Hilden, Germany) was equilibrated with 8 M urea in PBS, washed with the same buffer having 20 mM imidazole and the supernatant containing the protein was applied to it. The protein was eluted with elution buffer containing 8 M urea and 300 mM imidazole in PBS. The fractions containing the protein of interest were subjected to refolding. 1 ml of the protein containing fraction was added to 10 ml of refolding buffer [10% glycerol, 0.5% LDAO (N,N-Dimethyldodecylamine N-oxide)] and incubated overnight at 4 °C with constant stirring. The refolded protein was subjected to size exclusion chromatography using a Sephacryl S200 column (GE Healthcare, Piscataway, NJ, USA) equilibrated with buffer containing 10 mM Tris-Cl, 10 mM NaCl and 0.5% LDAO. The fractions were eluted using the same buffer and were analyzed by SDS-PAGE for protein and kept in -20 °C for further use.

### Limulus Ameobocyte Lysate Assay for estimation of endotoxin in purified protein preparation

Presence of endotoxin in protein preparations was measured by E-TOXATE™ Kit (Sigma Aldrich, St. Louis, MO) according to manufacturer’s instructions.

### Cell line and Culture conditions

The murine macrophage RAW 264.7 cells were cultured in RPMI 1640 media supplemented with 10% fetal bovine serum, 100 U/ml penicillin and 100 U/ml streptomycin (Invitrogen Life Technologies, Carlsbad, CA, USA), at 37 °C in a 5% CO_2_ humidified incubator. THP-1 cells were cultured under similar conditions as RAW 264.7 cells.

### Human PBMC isolation

Blood was drawn from a healthy donor and 1.5 mg EDTA (HiMedia, Mumbai) was added per ml blood drawn to prevent coagulation. Blood was diluted 1:1 with PBS. Diluted blood was layered over Histopaque-1077 (Sigma-Aldrich, St. Louis, MO, USA) in a ratio of 1:1 carefully to prevent mixing. The sample was centrifuged at 400 xg for 30 mins without acceleration or deceleration at room temperature. After centrifugation, the upper plasma layer was discarded and buffer layer was collected and transferred into new centrifuge tube. Buffy coat cells were washed twice with PBS and centrifuged at 250 xg for 10 mins at room temperature. Cells were resuspended in culture medium containing RPMI 1640 media supplemented with 10% fetal bovine serum, 100 U/ml penicillin and 100 U/ml streptomycin (Invitrogen Life Technologies, Carlsbad, CA, USA). Work with human blood has been approved by Institutional Bioethics Committee.

### Experimental design

For gene expression studies, cells were plated at a density of 2x10^6^ cells/ml in a 6 well plate with 2 ml of complete media in each well. The cells were treated with 5 µg/ml Polymyxin B sulphate (PmB) (Sigma-Aldrich, St. Louis, MO, USA) for 30 mins followed by treatment with OmpU (1.5 µg/ml) or *E. coli* LPS (1 µg/ml) (Sigma-Aldrich, St. Louis, MO, USA) and cells were harvested for RNA isolation at various time points.

To check expression levels of NO, TNFα and IL-6 at various time points, cells were plated in a 6 well plate with 1.5 ml of complete media at a density of 1x10^6^ cells/ml in each well. Cells which were to receive OmpU treatment were first treated with 5 µg/ml PmB for 30 mins followed by treatment with 1.5 µg/ml OmpU. Cells treated with LPS (1 µg/ml) served as a positive control. Alternatively, for dose dependent studies cells were plated similarly at a density of 1x10^6^ cells/ml in a 6 well plate with 1.5 ml complete media and treated with or without 5 µg/ml PmB for 30 mins followed by treatment with different doses of protein. Cells treated with LPS (1 µg/ml) served as positive control. Expression for various mediators in dose dependent studies were checked at particular time point as determined previously from the time course profiling. Purified OmpU in elution buffer containing 10 mM Tris-Cl, 10 mM NaCl and 0.5% LDAO was diluted in PBS containing 0.5% LDAO to yield appropriate working concentrations. Similarly, elution buffer diluted in PBS + 0.5% LDAO described as protein-buffer in the figure legends served as negative control for time course and dose dependent experiments. LPS was diluted in PBS for treatments.

For down-regulation studies, with the cell density parameters being same as above, cells were treated with 5 µg/ml PmB for 30 mins, after which OmpU was added and cells were incubated for 24 h. Cells were re-plated in fresh media without PmB and stimulated with LPS for the defined time period which was determined from the time course studies.

LPS by itself is unable to induce IL-12 production from human PBMCs. To induce IL-12 production, PBMCs (1x10^6^ cells/ml) were treated with 100 ng/ml recombinant IFNγ (Peprotech, Rocky Hill, NJ) and then re-plated in fresh media and challenged with 1 µg/ml LPS. Initially to find out the optimal incubation period for IL-12 stimulation, PBMCs were treated with 100 ng/ml IFNγ for 8 h, 16 h and 24 h and then re-plated in fresh media and challenged with 1 µg/ml LPS for 24 h. Supernatants were collected and analyzed for the presence of IL-12p70. Similarly, to find out the optimum time point for induction of IL-12 response with LPS challenge, in a separate set of experiments PBMCs were stimulated with IFNγ for 16 h and re-plated and challenged with LPS for 16 h or 24 h. Supernatants were collected and analyzed for the presence of IL-12p70.

For IL-12 down-regulation experiments, cells were treated with OmpU at different doses followed by treatment with 100 ng/ml recombinant IFNγ. After incubation for definite time period, cells were re-plated in fresh media without PmB and challenged with 1 µg/ml LPS. Supernatants were collected and analyzed for IL-12 production by IL-12p70 ELISA. Pre-incubation time period for OmpU was 24 h, incubation time with IFNγ treatment and LPS challenge was determined from time course experiments.

### Cell viability analysis by MTT assay

THP-1 cells were plated at a density of 1x10^6^ cells/ml in 100 µl complete media in a 96 well plate. Cells were treated with OmpU (1.5 µg/ml or 3 µg/ml) or protein-buffer for 24 h. PmB (5 µg/ml) was added to the culture 30 mins prior to the treatment. Cells treated with PmB only served as control. After 24 h incubation, MTT assay was performed. Briefly, MTT solution (Sigma-Aldrich) was added to culture so that the final concentration was 10% (10 µl to 100 µl culture). Cells were then incubated for 3 h. After incubation, 100 µl of acidified propanol (0.1 N HCl in isopropanol) was added to cultures until the MTT crystals dissolved. Absorbance was measured at 570 nm. For calculation purpose, cells with media alone (untreated cells) were considered as 100% viable and media without any cells (media blank) was considered as no viability. Percentage cell viability was calculated by the formula using absorbance values at 570 nm: (Treated cells – media blank) *100/ (Untreated cells – media blank).

For cell viability assessment in down-regulation studies, the design was similar as that mentioned above. But after 24 h treatments, cells were re-plated in 100 µl fresh media without PmB and stimulated with 1 µg/ml LPS and incubated for further 24 h. LPS was not added to control wells (untreated cells and media without any cells). MTT assay was performed as described above.

### Analysis of Nos2, Tnfα and Il6 mRNA

RNA isolation was carried out using Nucleo-pore RNA sure mini kit (Genetix Biotech, New Delhi, India) according to manufacturer’s instructions. cDNA was synthesized from the total RNA obtained using Maxima First Strand cDNA Synthesis Kit (Thermo, Fisher Scientific, Waltham, MA, USA). Semi-quantitative real time PCR was performed using Maxima SYBR Green qPCR Master Mix (Thermo, Fisher Scientific, Waltham, MA, USA) on Eppendorf Mastercycler EP Realplex Thermal Cycler (Eppendorf, Hamburg, Germany) according to the manufacturer’s protocol. Primer sequences for genes were sourced from Primer Bank [18]. Primers for specified genes were synthesized by IDT Technologies (Integrated DNA Technologies, Coralville, IA, USA). The real-time PCR data was analyzed by the comparative C_T_ method of Schmittgen and Livak [19].

### Measurement of NO production

Synthesis of NO in RAW 264.7 cells in response to OmpU and LPS stimulation was measured by Griess reagent (Sigma-Aldrich, St. Louis, MO, USA). Cells were cultured in RPMI 1640 phenol red free media with 10% FBS and were plated at a density of 1x10^6^ cells/ml in the same media and incubated for 2 h in the CO_2_ incubator at 37 °C prior to treatment with OmpU, LPS or both. After 24 h of incubation, culture supernatants were collected, mixed with an equal volume of 1X modified Griess reagent and incubated in dark at room temperature for 15 mins. The absorbance was measured at 540 nm on iMark Microplate Absorbance Reader (Bio-Rad Laboratories, Hercules, CA, USA) and nitrite levels were determined using standard curves generated using sodium nitrite (Sigma-Aldrich, St. Louis, MO, USA).

### Determination of TNFα, IL-6 and IL-12p70

Cytokines such as, TNFα, IL-6 and IL-12 in the culture supernatants were measured by sandwich ELISA. BD OptEIA ELISA kits (BD Biosciences, San Jose, CA, USA) were used and ELISA was performed according to the manufacturer’s protocol. The optical density of each well was determined using iMark microplate absorbance reader (Bio-Rad Laboratories, Hercules, CA, USA).

### Statistical analysis

Data is expressed as the mean ± SEM. Statistical analysis was performed using Student’s two-sided *t* test. Differences were considered statistically significant at *p* <0.05.

### Software used for preparation of figures

Figures were prepared using Origin from OriginLab and Adobe Photoshop.

## Results

### I- OmpU, an inducer of pro-inflammatory responses

#### OmpU induces low levels of NO production in RAW 264.7 murine macrophage cell line

**Figure 1:**
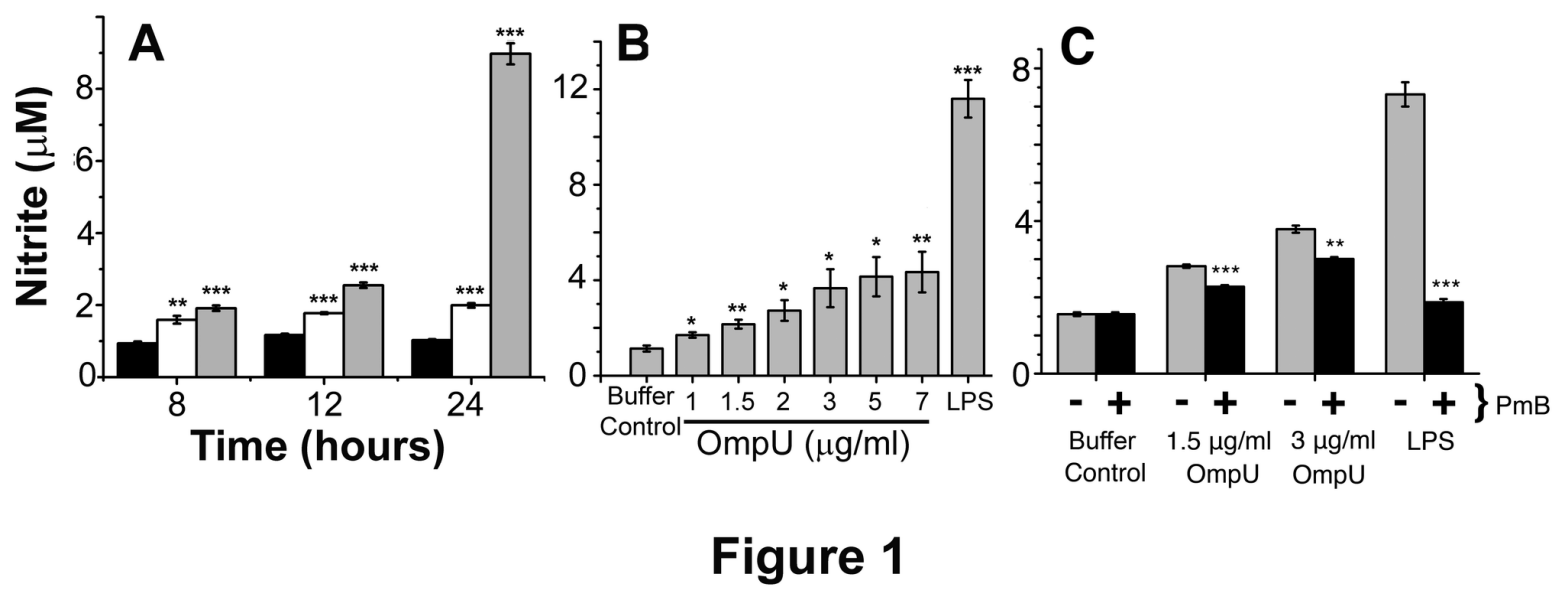
*V. cholerae* OmpU induces NO release from treated cells. RAW 264.7 murine macrophage cells were plated and treated with OmpU or LPS or protein-buffer (10 mM Tris-Cl, 10 mM NaCl, 0.5% LDAO diluted in PBS containing 0.5% LDAO). Polymyxin B (PmB) was added to the culture 30 mins prior to OmpU or buffer treatments. LPS and protein-buffer served as positive and negative control respectively in all experiments. Supernatants were collected and analyzed by Griess reaction for the production of nitrite, a stable end product of NO. Results are expressed as mean ± SEM and represent the average of three independent experiments. For Figure 1A and 1B, *p< 0.05, **p< 0.01 ***p < 0.001 versus buffer control. For Figure 1C, *p< 0.05, **p< 0.01 ***p < 0.001 versus non-PmB treatments. **A**. Time course experiments in response to OmpU shows considerable production of NO at 24 h in RAW 264.7 cells. **Black solid bar represents negative control; white solid bar represents OmpU and grey solid bar represents positive control**. **B**. NO levels in OmpU treated cells increase in a dose dependent manner with increasing doses of OmpU. Cells were treated with different doses of OmpU and incubated for 24 h. **C**. RAW 264.7 murine macrophage cells release slightly less NO when treated with OmpU along with PmB compared to cells treated with OmpU alone. RAW 264.7 cells were treated with buffer, 1.5 µg/ml and 3 µg/ml OmpU or 1 µg/ml LPS. Simultaneously, another set of cells were pre-treated with PmB and after 30 mins, cells were similarly treatmented. After 24 h, NO levels were assessed. **Gray bars represent cells without PmB treatment**. **Black bars represent cells with PmB treatment**.

NO released by macrophages upon recognition of PAMPs is an important early effector of the host innate immunity. A time course experiment for the NO release by RAW 264.7 murine macrophages in response to OmpU treatment was carried out (Figure 1A). RAW 264.7 cells were treated with 1.5 µg/ml of OmpU and supernatants were collected at 4 h, 8 h, 12 h and 24 h time points. At 4 h, NO was not detected but there was an increase in NO levels with increase in time. Further, cells were treated with different doses of OmpU, and supernatants were collected after 24 h incubation. It was observed that NO production induced by OmpU occurred in a dose-dependent manner (Figure 1B). Although, LAL test revealed that purified OmpU has no detectable endotoxin contamination (<0.06 EU), when we assessed NO levels in RAW 264.7 macrophages, we observed that NO production was slightly reduced upon PmB treatment (Figure 1C). Therefore, for further studies we used PmB along with the OmpU as mentioned in the method and result section.

#### OmpU treated cells produce TNFα

**Figure 2:**
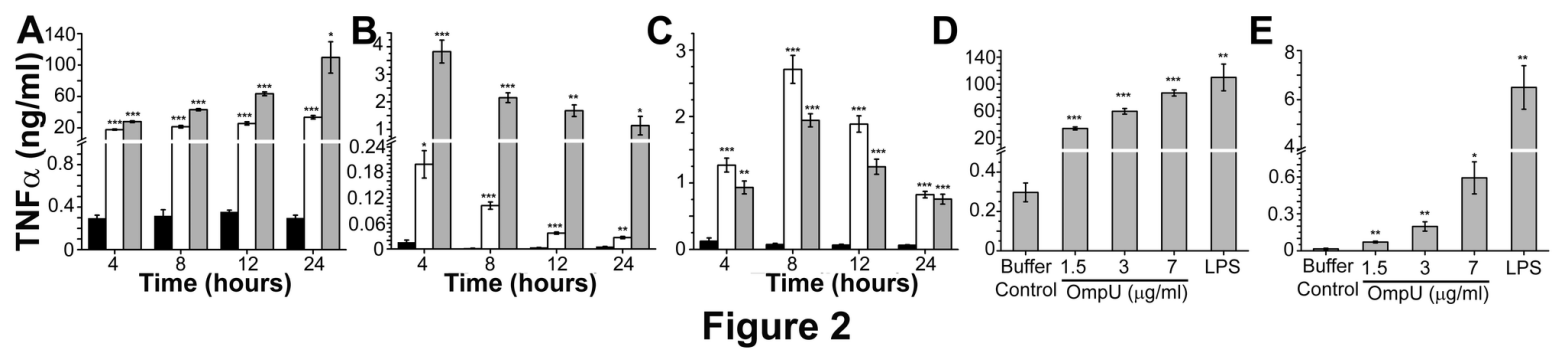
TNFα production in response to OmpU. RAW 264.7 murine macrophage cells, THP-1 human monocytic cells and human PBMCs were plated and treated with OmpU or LPS (1 µg/ml) or protein-buffer. PmB was added to the culture 30 mins prior to OmpU and buffer treatments. LPS and protein-buffer served as positive and negative controls respectively in all experiments. Supernatants were collected at various time points and analyzed for presence of TNFα by sandwich ELISA. Results are expressed as mean ± SEM and represent the average of three independent experiments. *p< 0.05, **p< 0.01 ***p < 0.001 versus buffer control. **Black solid bar represents negative control; white solid bar represents OmpU and grey solid bar represent positive control in Figure 2A, 2B and 2C**. **A**. A time dependent increase of TNFα was observed in RAW 264.7 cells in response to OmpU treatment. **B**. Time course experiments in THP-1 cells showed a time dependent decrease in TNFα production in response to OmpU treatment. **C**. Time course experiments in PBMCs showed a peak response of TNFα production at 8 h in response to OmpU treatment. **D**. Dose dependent increase in TNFα levels was observed in response to OmpU treatment in RAW 264.7 cells. Cells were treated with 1.5 µg/ml, 3 µg/ml and 7 µg/ml OmpU and incubated for 24 h. **E**. An increase in TNFα production was observed in THP-1 cells with increase in OmpU doses. Cells were treated with 1.5 µg/ml, 3 µg/ml and 7 µg/ml OmpU for 4 h.

TNFα is a pleiotropic cytokine which is produced by cells of monocytic lineage in response to an inflammatory stimulus. Its production in response to OmpU treatment was assessed in murine macrophage cell line RAW 264.7, human monocytic cell line THP-1, and PBMCs. RAW 264.7 macrophages were treated with 1.5 µg/ml OmpU, whereas THP-1 cells and human PBMCs were treated with 3 µg/ml OmpU. Generally, since the monocyte response is less than macrophage response, we chose a higher protein dose for treating monocytes. Supernatants were collected after 4 h, 8 h, 12 h and 24 h incubation periods. The peak response of TNFα differed in different cells. A gradual time-dependent increase in TNFα production was observed in RAW 264.7 cells treated with OmpU with maximum production at 24 h as seen in Figure 2A. In case of THP-1 cells the peak response was at 4 h (Figure 2B), whereas, in human PBMCs the peak response of TNFα production was observed at 8 h (Figure 2C). Dose-dependent increase of TNFα production was observed in response to increasing doses of OmpU (Figure 2D, 2E).

#### IL-6 release is induced in OmpU treated cells

**Figure 3:**
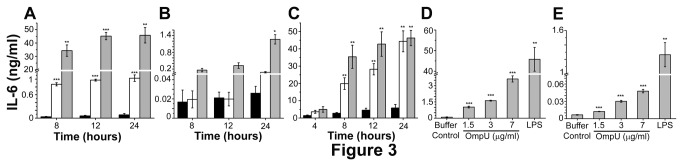
*V. cholerae* OmpU induce IL-6 production from treated cells. RAW 264.7 murine macrophage cells, THP-1 human monocytes and human PBMCs were plated and treated with OmpU or LPS or protein-buffer. PmB was added to the culture 30 mins prior to OmpU and buffer treatments. LPS and protein-buffer served as positive and negative controls respectively in all experiments. Supernatants were collected at various time points and analyzed for the presence of IL-6 by sandwich ELISA. Results are expressed as mean ± SEM and represent the average of three independent experiments. *p< 0.05, **p< 0.01 ***p < 0.001 versus buffer control. **Black solid bar represents negative control; white solid bar represents OmpU and grey solid bar represent positive control for Figure 3A, 3B and 3C**. **A**. A time dependent increase of IL-6 was observed in RAW 264.7 cells in response to OmpU treatment. **B**. A time dependent increase was observed in THP-1 monocytes in response to OmpU treatment. **C**. In human PBMCs, increased production of IL-6 was observed with increase in OmpU incubation. **D**. Dose dependent increase in IL-6 levels was observed in RAW 264.7 with increase in OmpU concentration. **E**. Dose dependent increase in IL-6 levels was observed in THP-1 monocytes with OmpU treatment.

The effect of OmpU on the IL-6 production was examined in RAW 264.7 cells, THP-1 cells and human PBMCs. IL-6 production increased in a time-dependent manner in murine macrophage RAW 264.7 cell line and THP-1 human monocytic cell line (Figure 3A and Figure 3B). Likewise, in human PBMCs, IL-6 response to OmpU increased with time (Figure 3C). IL-6 was maximally produced at 24 h across all the cell types. Further, we observed that the IL-6 levels increased with OmpU doses (Figure 3D and Figure 3E).

#### II: OmpU suppresses the LPS-mediated inflammatory response

Our initial findings demonstrated that macrophage cell line RAW 264.7 produced NO, TNFα and IL-6 (Figure 1, Figure 2, and Figure 3), while human monocytic cell line THP-1 and human PBMCs produced TNFα and IL-6 (Figure 2, Figure 3) in response to OmpU. Further we performed time course experiments with 1 µg/ml LPS. We observed maximal release of NO in response to LPS at 24 h (Figure 1A). TNFα production in response to LPS was considerable at 24 h in RAW 264.7 cells (Figure 2A), at 4 h in THP-1 (Figure 2B), and at 8 h in human PBMCs (Figure 2C). Substantial production of IL-6 in response to LPS was observed at 24 h for RAW 264.7 (Figure 3A), 24 h for THP-1 (Figure 3B) and 24 h for PBMCs (Figure 3C).

Furthermore, we observed that pre-treatment with OmpU suppressed the LPS-mediated responses. The extent and pattern of down-regulation varied in different cell types. Cells were pre-treated with OmpU for 24 h after which they were re-plated in fresh media and stimulated with 1 µg/ml LPS for definite time determined from time course experiments (Figure 1A, Figures 2A, 2B, 2C, Figure 3A, 3B, 3C).

**Figure 4:**
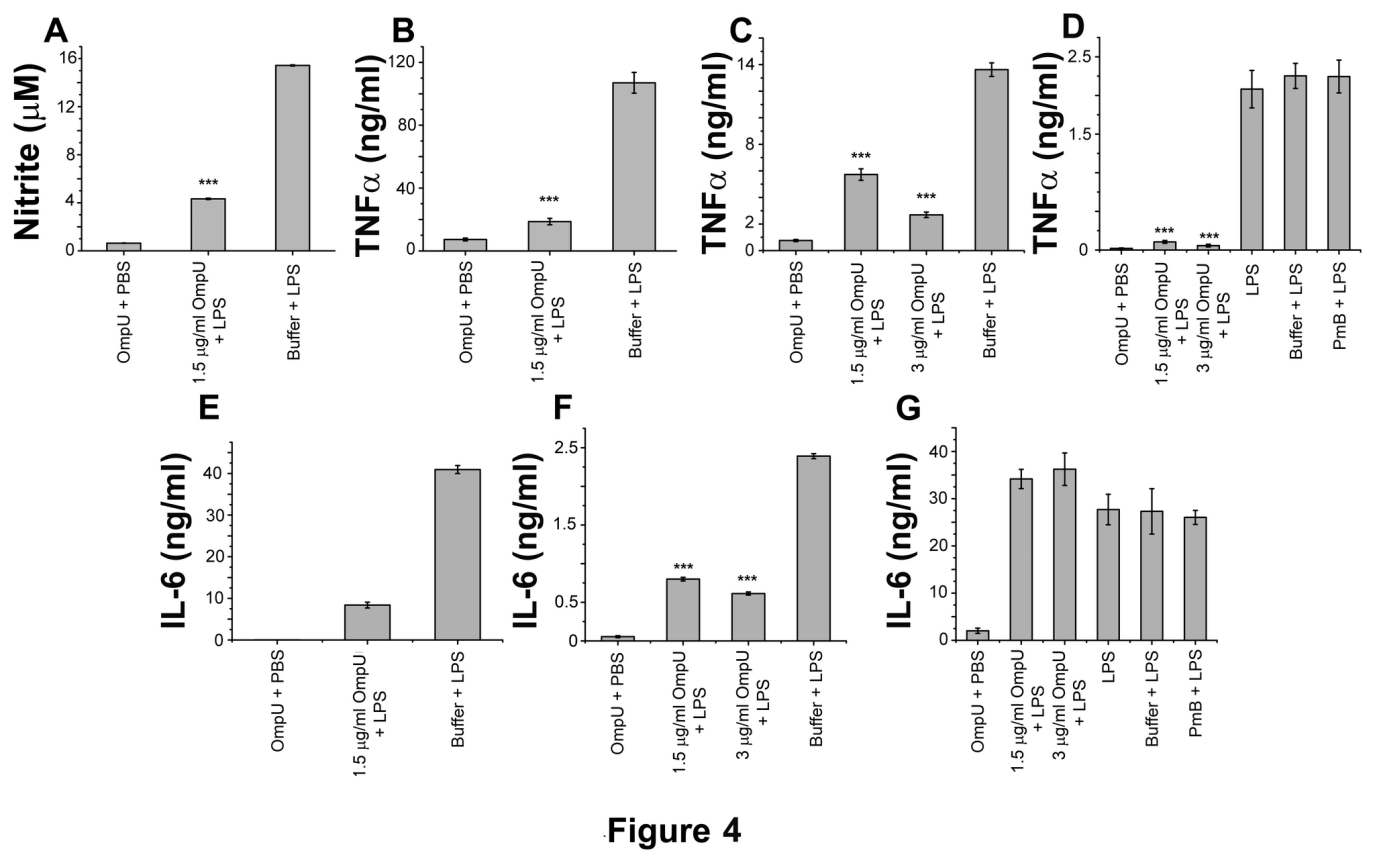
Pre-treatment of cells with OmpU suppresses LPS mediated pro-inflammatory responses. RAW 264.7 murine macrophage cells, THP-1 human monocytes and human PBMCs were plated and treated with OmpU or protein-buffer and incubated for 24 h. PmB was added to the culture 30 mins prior to the treatment. After 24 h of OmpU or buffer treatment, cells were re-plated in fresh media without PmB and challenged with LPS for defined time period at which LPS induced response was maximum for mediator of interest. Control experiments were performed to evaluate the contribution of buffer or polymyxin B (PmB) for OmpU mediated down-regulatory phenomenon. Supernatants were collected and analyzed for the presence of pro-inflammatory mediators. Nitrite production was estimated by Griess reaction. TNFα and IL-6 levels were evaluated by sandwich ELISA. Results are expressed as mean ± SEM and represent the average of three independent experiments. *p< 0.05, **p< 0.01 ***p < 0.001 versus protein buffer + LPS control. **A**. OmpU petreatment down-regulated NO production in LPS stimulated RAW 264.7 cells. After 24 h of protein-buffer or OmpU (1.5 µg/ml) pre-treatment, cells were re-plated in fresh media and stimulated with 1 µg/ml LPS for further 24 h. Down-regulation of NO was as much as 75%. **B**. RAW 264.7 cells pre-treated with OmpU, showed down-regulation of LPS mediated TNFα production. **C**. Pre-treatment with OmpU down-regulated LPS mediated TNFα production in THP-1 monocytic cells. An increase in down-regulation of LPS induced TNFα production was observed with increase in OmpU concentration. **D**. In human PBMCs, an increase in down-regulation of LPS induced TNFα occurred with increase in OmpU doses. **E**. Down-regulation of LPS mediated IL-6 production was observed in RAW 264.7 cells pre-treated with OmpU. **F**. In THP-1 human monocytes an increase in down-regulation of LPS induced IL-6 occurred with increase in OmpU doses. **G**. OmpU pre-treatment did not suppress LPS induced IL-6 production in human PBMCs.

Pre-treatment with 1.5 µg/ml OmpU significantly decreased LPS-induced nitrite production (Figure 4A; p< 0.001) in RAW 264.7 cell line. Similarly, pre-treatment with 1.5 µg/ml OmpU was sufficient to suppress TNFα production in response to LPS by 85-91% (p < 0.001; Table 1) in RAW 264.7 cell line (Figure 4B; Table 1), 55-61% (p < 0.001; Table 1) in THP-1 cell line (Figure 4C; Table 1), and 94-96% (p < 0.001) in human PBMCs (Figure 4D; p< 0.001; Table 1). Dose-dependent suppression of TNFα production was observed in THP-1 and PBMCs with increase in OmpU pre-treatment doses (Table 1, Figure 4C; (p < 0.001) and Figure 4D (p < 0.001)). Moreover, OmpU pre-treatment also suppressed the IL-6 response of LPS-treated cells. Pre-incubation with 1.5 µg/ml OmpU suppressed IL-6 by 77-83% in RAW 264.7 cell line (Figure 4E; p < 0.001; Table 2). In THP-1 cell line, pre-treatment with 1.5 µg/ml OmpU suppressed IL-6 production by 63-69% in LPS-activated cells (Figure 4F, p < 0.001; Table 2). Dose-dependent suppression of IL-6 with increasing doses of OmpU was observed in THP-1 cell line (Figure 4F; p < 0.001; Table 2). IL-6 production in LPS-induced PBMCs was not suppressed by OmpU pre-treatment (Figure 4G).

**Table 1:** Percentage of suppression of LPS mediated TNFα production in OmpU pre-treated cells.

**Cell type**	**OmpU Dose (µg/ml)**	**Percentage of down-regulation**	**p value**
RAW 264.7	1.5	85-91%	0.0002
THP-1	1.5	55-67%	0.0003
	3	80-88%	0.0001
Human PBMCs	1.5	94-96%	0.0001
	3	96-98%	0.0001

**Table 2:** Percentage of suppression of LPS mediated IL-6 production in OmpU pre-treated cells.

**Cell type**	**OmpU Dose (µg/ml)**	**Percentage of down- regulation**	**p value**
RAW 264.7	1.5	77-83%	0.0001
THP-1	1.5	63-69%	0.0001
	3	73-75%	0.0001

**Figure 5:**
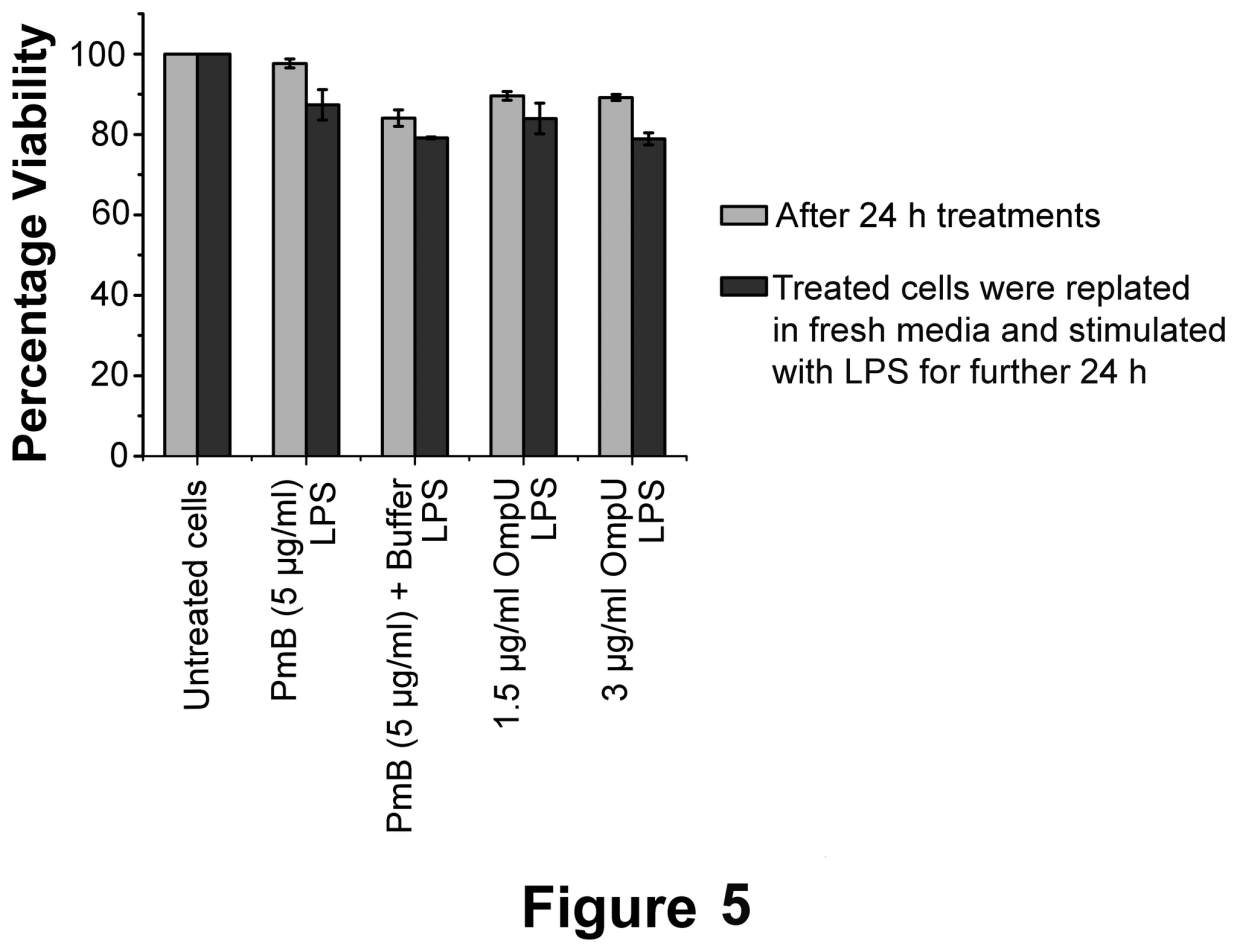
Assessment of Cell health by MTT Assay. THP-1 human monocytes were plated and treated with OmpU or protein-buffer or PmB and incubated for 24 h and cell viability was measured by MTT assay. After 24 h of same treatment, cells were re-plated in fresh media without PmB and challenged with LPS for 24 h. Cell viability was measured by MTT assay after 24 h of LPS treatment. It was observed that cell health was minimally affected during down-regulation experiment. Results are expressed as mean ± SEM and represent the average of three independent experiments.

MTT assay was performed to check whether the down-regulation of various inflammatory mediators was due to deterioration in cell health (Figure 5). We observed that cell viability was minimally affected across different treatments and incubation periods.

#### III: Differential regulation of pro-inflammatory cytokine occurs in the gene level

To determine whether the differential regulation of OmpU occurred at the level of gene expression, mRNA expression of *Nos2*, *Tnfα*, *Il6* in RAW 264.7 cells was determined by semi-quantitative RT-PCR.

**Figure 6:**
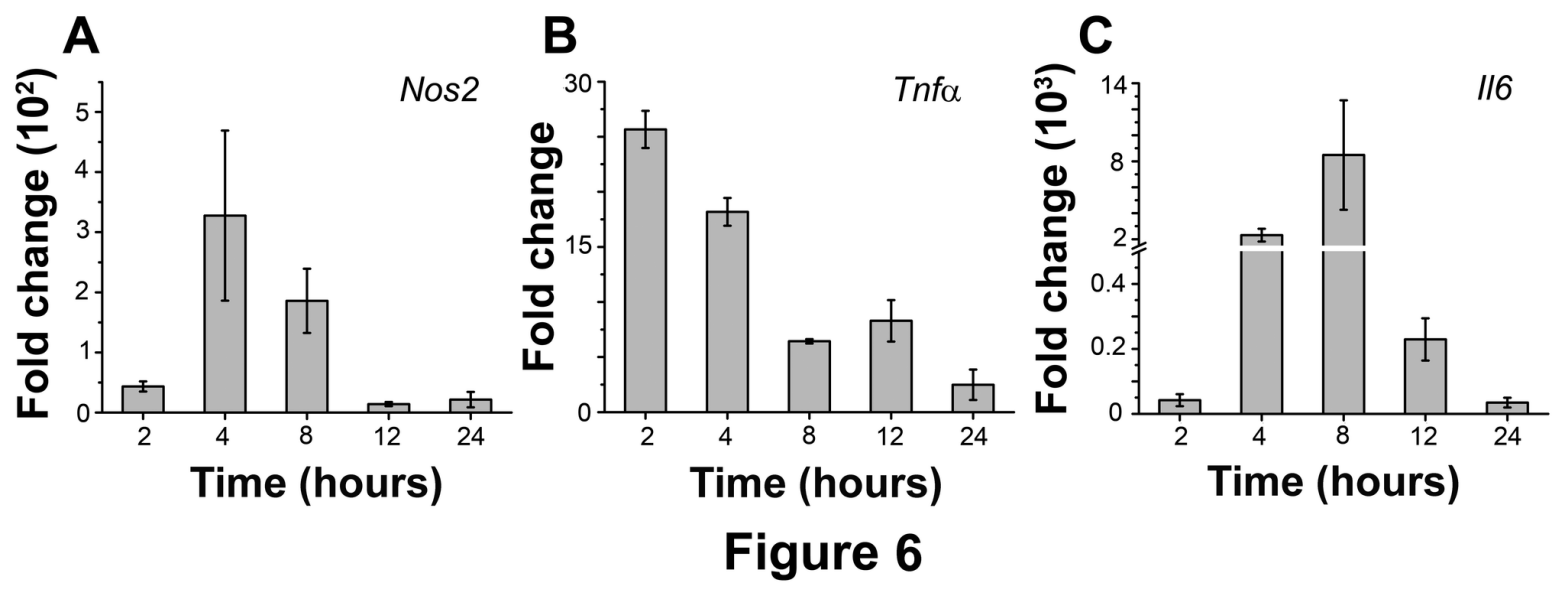
Time course profiling of *Nos2*, *Tnfα* and *Il6* mRNA expression in response to OmpU in RAW 264.7 cells. RAW 264.7 cells were plated and treated with OmpU or protein-buffer. Cells were harvested after 2 h, 4 h, 8 h, 12 h and 24 h incubation periods and total RNA was isolated. Semi quantitative RT-PCR was performed to evaluate changes in mRNA expression of *Nos2*, *Tnfα* and *Il6*. Results are expressed as mean ± SEM and represent the average of three independent experiments. **A**. Time dependent expression of *Nos2*. Maximum fold change of *Nos2* occurred at 4 h. **B**. Time dependent expression of *Tnfα*. Maximum fold change of *Tnfα* occurred at 2 h. **C**. Time dependent expression of *Il6*. Maximum fold change of *Il6* occurred at 8 h.

**Figure 7:**
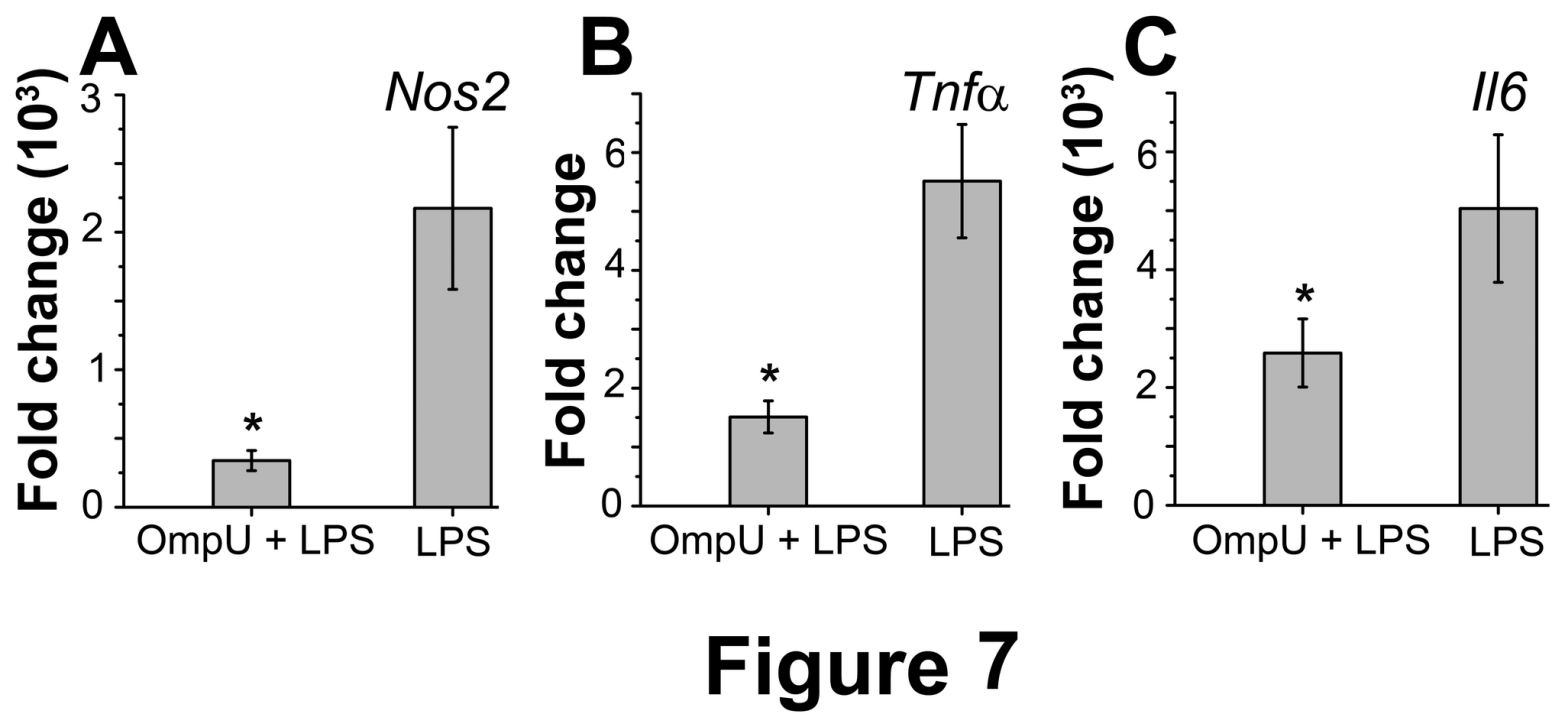
Suppression of LPS mediated of *Nos2*, *Tnfα* and *Il6* mRNA expression in OmpU pre-treated RAW 264.7 cells. RAW 264.7 cells were plated and treated with OmpU or protein-buffer and incubated for 24 h. After 24 h of OmpU or protein-buffer treatment, cells were re-plated in fresh media without PmB and challenged with LPS and incubated for 8 h. Cells were harvested and total RNA was isolated. Semi quantitative RT-PCR was performed to evaluate changes in mRNA expression of *Nos2*, *Tnfα* and *Il6*. Results are expressed as mean ± SEM and represent the average of three independent experiments. *p< 0.05, **p<0.01 ***p < 0.001 versus LPS. **A**. Suppression of LPS mediated *Nos2* expression was observed in RAW 264.7 cells pre-treated with OmpU. **B**. *Tnfα* expression was suppressed in LPS activated RAW 264.7 cells pre-treated with OmpU. **C**. OmpU pre-treatment suppressed LPS mediated of *Il6* expression in RAW 264.7 cell line.

Nitric oxide synthase, encoded by *Nos2* gene mediates the synthesis of NO. The expression profile of this gene in response to OmpU treatment for various time periods is shown in Figure 6A. It was observed that the maximum induction in gene expression took place at 4 h (327 fold change), whereas a minimum induction (13.9 fold change) was observed at 12 h. Time course experiments for *Nos2* gene up-regulation in response to 1 µg/ml LPS was also carried out (data not shown). The effect of OmpU pre-treatment on LPS induced expression of *Nos2* gene was also ascertained (Figure 7A). As observed in case of NO, OmpU pre-treatment reduced the LPS induced up-regulation in *Nos2* gene by 84% (p< 0.01, Figure 7A).

The change in *Tnfα* gene up-regulation brought about by OmpU treatment was maximum at 2 h (25 fold) which decreased to 2.5 fold at 24 h (Figure 6B). The effect of pre-treatment of RAW 264.7 murine macrophage cell line with OmpU on LPS induced expression of *Tnfα* expression was also assessed (Figure 7B). OmpU pre-treatment with RAW 264.7 cells decreased TNFα mRNA levels in response to LPS as much as 72% (p <0.01, Figure 7B).

At the gene level, it was observed that OmpU treatment brought about a maximum 8500 fold up-regulation of *Il6* gene at 8h (Figure 6C). The down-regulatory effect of OmpU pre-treatment on the LPS induced *Il6* gene expression was also studied. Up-regulation induced by LPS treatment was reduced by 50% when cells were pre-treated with OmpU for 24 h (p < 0.05, Figure 7C).

#### IV: OmpU suppresses IL-12 production by stimulated human PBMCs

**Figure 8:**
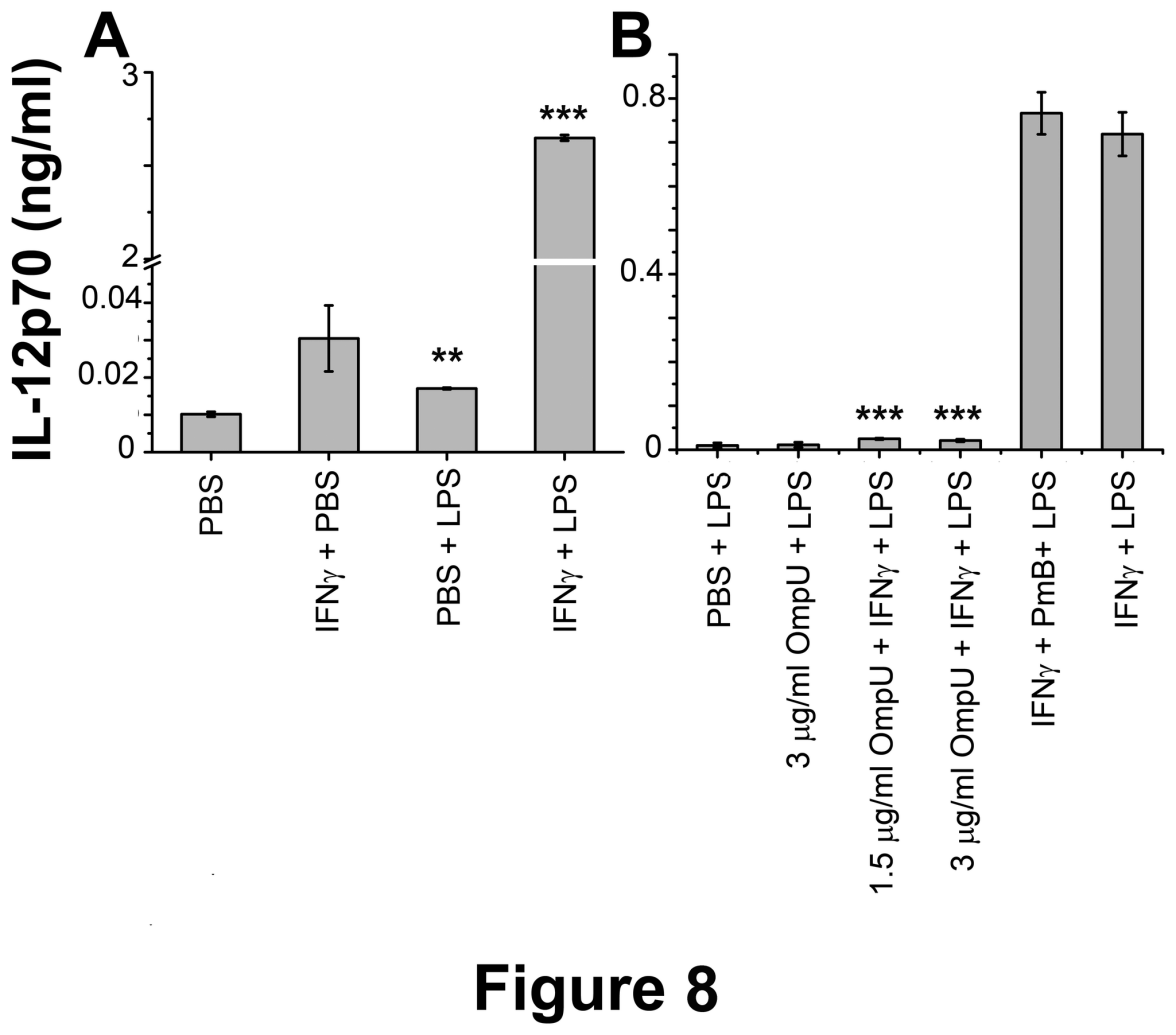
OmpU pre-treatment suppresses LPS mediated IL-12 production by human PBMCs. **A**. IL-12 production was induced in human PBMCs with IFNγ and LPS. Cells stimulated with IFNγ alone or LPS alone did not show IL-12 production. Results are expressed as mean ± SEM and represent the average of two independent experiments. **p < 0.01, ***p< 0.001 versus PBS. **B**. Down-regulation of LPS mediated IL-12 production was observed in human PBMCs pre-treated with OmpU. Cells were treated with various doses of OmpU. PmB was added to the culture 30 mins prior to the treatment. After 8 h of OmpU treatment, cells were treated with IFNγ. After 24h of OmpU treatment (16 h of IFNγ incubation), cells were re-plated in fresh media without PmB and challenged with LPS and further incubated for 24 h. A suppression of as much as 95% of LPS mediated IL-12p70 was seen across all doses. Results are expressed as mean ± SEM and represent the average of three independent experiments. ***p < 0.001 versus IFNγ+PmB+ LPS.

IL-12 is a known regulator of Th1 and Th2 responses and it also regulates innate immune responses. The possibility of OmpU mediated suppression of LPS induced IL-12 production was investigated in human PBMCs. PBMCs do not produce IL-12 in response to LPS treatment. To induce IL-12 production, cells were stimulated with 100 ng/ml IFNγ and then challenged with 1µg/ml LPS. From control experiments, we observed that optimal IL-12 production occurred when PBMCs were stimulated with IFNγ for 16 h followed by LPS challenge for 24 h. Therefore, the PBMCs were stimulated with IFNγ for 16 h, re-plated in fresh media without PmB and challenged with LPS for 24 h. IL-12p70 was evaluated in culture supernatants (Figure 8A). For down-regulation experiment, PBMCs (1x10^6^ cells/ml) were treated with 1.5 µg/ml and 3 µg/ml OmpU followed by IFNγ at 8 h, and then after 16 h of incubation cells were re-plated in fresh media and challenged with 1 µg/ml LPS for 24 h. A complete suppression of IL-12 production by PBMCs was observed when the cells were pre-incubated with as low as 1.5 µg/ml OmpU (Figure 8B).

## Discussion

Porins are the essential components of the outer membrane of gram-negative bacteria and are involved in multiple processes involved in bacterial homeostasis such as nutrient transport, antimicrobial resistance and responses to environmental signals. Furthermore, porins from various gram-negative species are involved in virulence processes such as adherence to the host cells and invasion [5,20-22]. In addition, some porins have the ability to act as PAMPs, and are recognized by TLRs to initiate downstream signaling cascades resulting in anti-microbial responses in the host [23-28].

OmpU, one of the major outer membrane porins of *Vibrio* species, is involved in several host-pathogen interactions. Many functions of OmpU in *V. cholerae* are linked to ToxR regulon. ToxR regulon, a master virulence gene controller, regulates the expression of cholera toxin, toxin co-regulated pilus as well as accessory colonization factors which together work towards establishing the bacteria in the human gut [29]. OmpU expression is positively regulated by the ToxR regulon. A homologue of the ToxR regulon also exists in *V. parahaemolyticus* [30].

In both these species, up-regulation of OmpU by ToxR regulon or its homologue helps in survival of the pathogen in the intestine by providing resistance against bile salts and acids [30-33]. Further, *V. cholerae* OmpU has the ability to confer resistance against anti-microbial peptides [34]. This property of host anti-microbial peptide resistance is shared by OmpU with another *Vibrio* species, *V. splendidus*, a pathogen which infects oyster ( *Crassostrea* *gigas* ) [35].

OmpU from several *Vibrio* species have been implicated in adherence to host cells. OmpU of *V. splendidus* is involved in attachment to oyster hemocytes and is one of the key factors for cellular invasion [20]. OmpU of human pathogen *V. vulnificus* possesses the ability to bind to fibronectin [36]. There have been contradictory reports regarding the role of *V. cholerae* OmpU as an adherence factor to the host cell. Kaper et al [14], suggested that *V. cholerae* OmpU might act as an adhesin, but later Iwanagai et al [37], reported that OmpU does not have adhesive properties. Recently, Sarkar et al [38], reported that *V. cholerae* OmpU contributes to IL-8 production possibly by binding to epithelial cells.

The expression of OmpU in *V* *. alginolyticus* , which infects humans, fish and crustaceans, increases in presence of certain antibiotics like tetracycline and kanamycin, suggesting OmpU mediates antibiotic resistance to some extent [39].

OmpU from a few species of *Vibrio* have been evaluated as vaccine candidates. OmpU from *V* *. alginolyticus* has shown vaccine potential in *Lutjanus* *erythropterus* [40]. Further, OmpU from *V. harveyi* has been successfully isolated as a vaccine candidate in *Scophthalmus* *maximus* [41]. All these findings make *Vibrio* OmpU an interesting molecule for immunological study.

To explore whether *V. cholerae* OmpU possesses pro-inflammatory nature, we evaluated some important innate immune response mediators like NO, TNFα and IL-6. Macrophages produce NO to kill or inhibit the growth of invading microorganisms. Synthesis of NO in macrophages is mediated by inducible nitric oxide synthase (iNOS) in response to cytokines or pathogen-derived molecules. One such cytokine that induces iNOS is TNFα whose production is initiated via the TLR signaling pathway in response to various stimuli. TNFα signals macrophages to produce NO for destruction of bacteria and further localized signaling involved in inflammation [42]. It induces expression of chemokines and cell adhesion molecules in nearby endothelial cells, which promote recruitment of neutrophils and other leukocytes to the site of infection [43]. IL-6, another cytokine secreted by activated macrophages, is a well-known mediator of fever and acute phase response of innate immunity [44]. Apart from these effects, IL-6 promotes differentiation of B cells into plasma cells and proliferation of T cells [45,46]. As in the case of TNFα, IL-6 expression is also induced by recognition of PAMPs by the TLRs.

The effect of *V. cholerae* OmpU on the induction of key inflammatory mediators such as, NO, TNFα and IL-6 was investigated initially using RAW 264.7 murine macrophage cell line and THP-1 human monocytic cell line (Figure 1, Figures 2A, 2B, 2D, 2E, Figures 3A, 3B, 3D, 3E). Endotoxin level in purified protein preparation was found to be less than 0.06 EU/ml. Yet PmB was added to the culture prior to OmpU treatment to neutralize possible endotoxin contamination (Figure 1C). To explore whether pro-inflammatory effects of OmpU was only limited to cell lines of human and mouse origin, we investigated the effect of OmpU on human PBMCs (Figure 2C, Figure 3C). Our observations proved that OmpU has the potential to induce pro-inflammatory signals and behaves similarly to various other gram-negative bacterial porins in this aspect.

Several studies report differential regulation of pro-inflammatory cytokines by various molecules of pathogen and host origin. For instance, LPS, a very potent pro-inflammatory agent from gram-negative outer membrane can suppress its own effect when cells are pre-treated with LPS [47]. CtxB subunit of cholera toxin induces TNFα production as well as down-regulates TNFα, IL-6 and IL-12 [48-50]. Another agent of host origin, adiponectin, a glucocorticoid hormone has the ability to exhibit both pro-inflammatory and anti-inflammatory effects [51].

In our study, experiments were performed to determine whether OmpU could differentially regulate pro-inflammatory cytokines. The effect of OmpU pre-treatment was analyzed in LPS-activated cells in terms of suppression or down-regulation of the cytokine expression. OmpU pre-treated RAW 264.7 cells, THP-1 human cells and human PBMCs showed a significant decrease in the production of NO, TNFα, IL-6 and IL-12 in these respective cell types (Figure 4, Figure 8B) with the exception of IL-6, which was not down-regulated in human PBMCs (Figure 4G). Therefore, these observations suggest that OmpU has the ability to suppress LPS mediated effects, but it may not be able to suppress different pro-inflammatory mediators in different cell types.

It is known that cells pre-treated with LPS become tolerant to subsequent LPS stimulation. Though purified protein preparation has almost no detectable endotoxin contamination (EU< 0.06), as revealed by the LAL assay, we observed that there is a slight increase in NO production in OmpU treated cells as compared to PmB and OmpU treated cells (Figure 1C). To avoid LPS mediated tolerance, as a precautionary measure for down-regulation experiments, we used PmB along with OmpU treatment. After 24 h of incubation, cells were re-plated in fresh media without PmB and stimulated with LPS. Pre-treatment of cells with PmB alone did not suppress the effect of LPS (Figure 4D, Figure 8B). These findings suggest that OmpU exhibits immunosuppressive activities.

Cells can become non-responsive under stress or if the cell health is compromised. Since cells were treated with OmpU and further with LPS, we wanted to rule out the possibility that the down-regulation of inflammatory mediators was due to decreased cell viability. Hence, cell viability was assessed by MTT assay. Results showed that cell health did not significantly worsen upon various treatments and incubation periods (Figure 5). Therefore, the suppression of LPS mediated effects was not due to poor cell health.

Since immunosuppressive functions are attributed to cytokines, like IL-10 and TGFβ, the production of these two cytokines in response to OmpU was investigated. Our initial investigations carried out in RAW 264.7 cell line showed no evidence of IL-10 or TGFβ upon treatment with OmpU (data not shown).

We are currently investigating the mechanism of OmpU mediated differential regulation. The observed down-regulation in our study can be due to tolerance phenomenon. The anti-inflammatory effects of LPS and adiponectin were indeed attributed to the induction of tolerance due to the production of a pro-inflammatory signal; a phenomenon which may be true in our case as well. Tolerance induction by LPS and adiponectin involves multiple signaling pathways which ultimately alters in NFκB or AP-1 activation [52]. Cholera toxin, which is also similar to OmpU in terms of induction of pro-inflammatory as well as anti-inflammatory responses, induces its down-regulatory effect by activating MAP kinase phosphatase-1 or MKP-1, thus inhibiting MAP kinase signaling involved in cytokine biogenesis [53]. Study of MAP kinase signaling and regulation of transcription factors such as NFκB and AP-1 may reveal the nature and mechanism of anti-inflammatory responses triggered by *V. cholerae* OmpU.

## Conclusion

In sum, our study reveals that purified *V. cholerae* OmpU possesses dual nature of host immunomodulation. It can induce pro-inflammatory responses but on the other hand it has the potential to suppress the innate immune response at the early step of infection. Furthermore, since OmpU pre-treatment suppresses IL-12 production upon LPS stimulation, OmpU might have the ability to modulate T cell response as well.
