# Correction: *Vibrio cholerae* Porin OmpU Induces Pro-Inflammatory Responses, but Down-Regulates LPS-Mediated Effects in RAW 264.7, THP-1 and Human PBMCs

**DOI:** 10.1371/annotation/295cad0c-032f-407a-9e08-f84dea0491b1

**Published:** 2013-11-13

**Authors:** Sanica C. Sakharwade, Praveen K. Sharma, Arunika Mukhopadhaya

The third sentence of the subsection "Purification of recombinant OmpU" in Material and Methods refers incorrectly to *V. cholerae*. The corrected sentence is: *E. coli* was cultured in Luria broth (HiMedia, Mumbai, India) until the OD600 reached 0.5-0.6. 

